# Differences in the efficacy of climate forcings explained by variations in atmospheric boundary layer depth

**DOI:** 10.1038/ncomms11690

**Published:** 2016-05-25

**Authors:** Richard Davy, Igor Esau

**Affiliations:** 1 Nansen Environmental and Remote Sensing Center and Bjerknes Centre for Climate Research, Thormøhlensgt. 47, 5006 Bergen, Norway

## Abstract

The Earth has warmed in the last century and a large component of that warming has been attributed to increased anthropogenic greenhouse gases. There are also numerous processes that introduce strong, regionalized variations to the overall warming trend. However, the ability of a forcing to change the surface air temperature depends on its spatial and temporal distribution. Here we show that the efficacy of a forcing is determined by the effective heat capacity of the atmosphere, which in cold and dry climates is defined by the depth of the planetary boundary layer. This can vary by an order of magnitude on different temporal and spatial scales, and so we get a strongly amplified temperature response in shallow boundary layers. This must be accounted for to assess the efficacy of a climate forcing, and also implies that multiple climate forcings cannot be linearly combined to determine the temperature response.

## Abstract (web summary)

Correctly detecting surface temperature responses to a climate forcing requires consideration of their spatio-temporal variability. Here, the authors demonstrate that forcing efficacy is governed by the effective heat capacity of the atmosphere, itself a function of planetary boundary layer depth.

## Graphical abstract

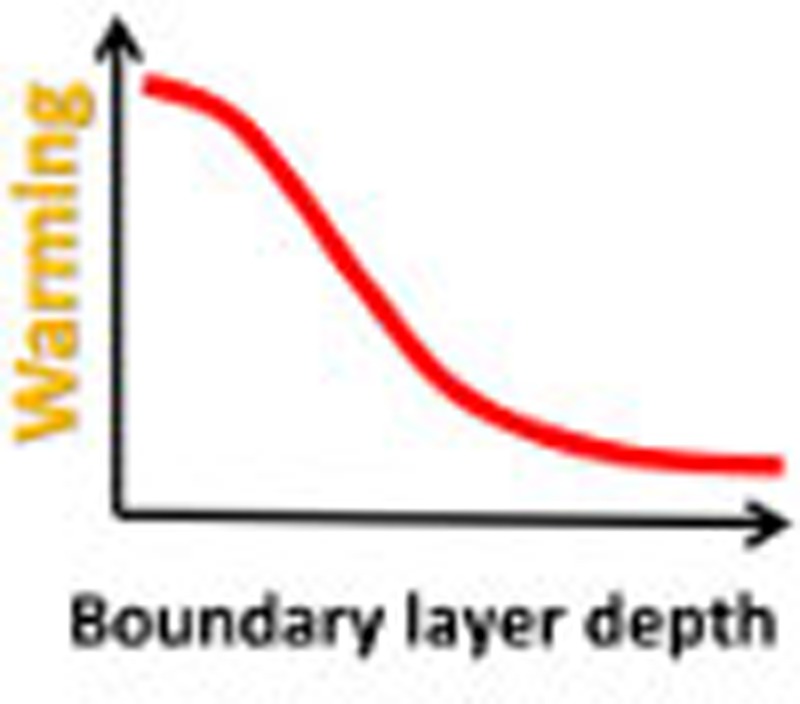

The detection of processes that affect the surface climate is one of the fundamental tools in our understanding of climate change. The assessment of climate-forcing processes is essentially a statistical, signal-to-noise problem. The most famous example is the detection of warmer surface air temperatures (SATs) attributed to the human-induced enhanced concentration of atmospheric carbon dioxide^1^,^2^. One of the challenges in the detection of climate forcing signals has been the strong spatial correlation between the strength of the surface temperature trends and the strength of natural variability^3^. This relation leads to an inherently low signal-to-noise ratio, regardless of the strength of temperature trends. Indeed, despite the well-established rapid warming in the Arctic^4^,^5^,^6^, the polar regions were the last part of the globe for which there was a successful detection and attribution of the recent warming to anthropogenic-enhanced greenhouse gas (GHG) concentrations^7^.

In addition to the detection of the influence of enhanced GHGs on surface temperatures, there have been numerous studies assessing how clouds^8^,^9^,^10^,^11^,^12^, precipitation^8^,^12^,^13^ and soil moisture^8^,^12^ may have introduced some of the observed temporal and regional variations to the overall warming trend that has been seen in the latter half of the twentieth century. In all these studies, the authors have adopted the commonly accepted linear regression model for establishing the relationship between a change in a given property and the temperature response^14^. However, it has been established from energy-budget models of the climate that temperature changes are linearly related to changes in the surface heating, but also inversely related to the effective heat capacity of the system^15^,^16^. Thus, although a linear-model approximation may work well under constrained conditions, such as focusing on a given region or season, in more wide-reaching studies it becomes necessary to account for variations in the effective heat capacity of the atmosphere. In recent times, it has also been recognized that the atmospheric convective mixing may have a strong effect on the observed and simulated climate through modulation of the low cloud and lapse-rate feedbacks^17^.

As the effective heat capacity is defined by the volume of air through which the heat is mixed, it can be related to the depth of the atmospheric boundary layer^18^. If we consider the one-dimensional energy budget of the lower atmosphere, then we define the change in SAT as being linearly related to the forcing and feedbacks in the climate system, and inversely related to the effective heat capacity of the system^19^, such that:


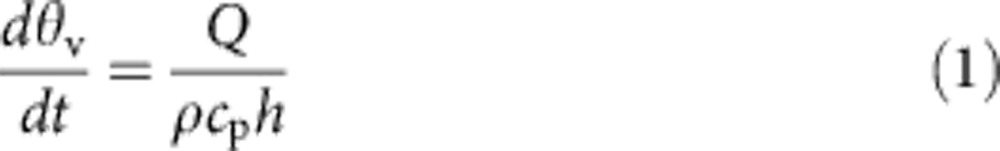


where *Q* (W m^−2^) is the heat flux divergence across a boundary layer of depth *h* (m), *ρ* (kg m^−3^) is the air density, *c*_p_ (J kg^−1^ K^−1^) is the heat capacity at constant pressure and *θ*_v_ (K) is the virtual potential temperature that is representative of the boundary layer. Henceforth, *θ*_v_ is taken at a height of 2  m above the ground. If differences in the effective heat capacity of the system are relatively small, we can linearly relate any changes to the heating to a change in *θ*_v_. However, if there are large variations in the effective heat capacity, we need to account for the dependency of the temperature response on the effective heat capacity. This is especially important when the effective heat capacity is small, as this can strongly magnify the strength of the SAT response to a perturbation in the surface energy budget^3^.

The hypothesis we put forward here presumes that there is essentially a decoupling of the planetary boundary layer (PBL) from the rest of the atmosphere. Such a decoupling may not be intuitive, but it is a rather natural concept, supported by numerous direct observations^20^,^21^, and indeed it is embedded in the PBL schemes of many (if not all) climate models^22^.

Consider the case of the urban PBL. The urban PBL is better mixed than the PBL in the rural background due to high surface roughness in the urban environment. Nevertheless, even the urban PBL shows a rather clear top boundary, separating it from the rest of the atmosphere^23^,^24^, which can be visualized by water vapour (Fig. 1). The well-known urban heat island effect^25^, which in northern cities may reach up to 10  K (ref. 26), demonstrates how heat may be trapped inside the urban boundary layer.

Now consider in this urban context, which can be so clearly visualized, the presumption that this decoupling is valid on climatological time scales. The urban heat island is highly variable in time and may appear for only a few hours in a given day. However, as the PBL effect is strongly selective and nonlinear, the average over climatological time scales is accumulating. We cannot state that the maximum effect will be observed over the time scale of 30–40 years, as addressed in this study, but over this period of time it is sufficiently large as well. Figure 2 illustrates the urban heat island, that is, the additional anthropogenic heat trapped in by the shallow PBL in Khanty–Mansiysk, according to MODIS satellite data analysis for 2000–2014. Thus, from numerous local studies, it has been firmly established that the PBL depth effect does exist. Our study here is the first to quantify this effect globally, using available data sets and models.

Different climate forcing processes such as variations in solar forcing, GHGs and aerosols have different efficacies in affecting the SAT^27^,^28^,^29^. In this work we have demonstrated that it is the variations in the effective heat capacity of the atmosphere, defined by the PBL depth, which can explain these differences in climate forcing efficacy. We must therefore question the assumption that different climate forcings are linearly additive in nature. This work highlights the pressing need to obtain a robust physical climatology of the boundary layer depth from the observational network and to be able to simulate this climatology with global climate models, to better constrain our estimates of surface climate response to climate forcings.

## Results

### Temperature trend amplification in shallow boundary layers

**Figure 3:**
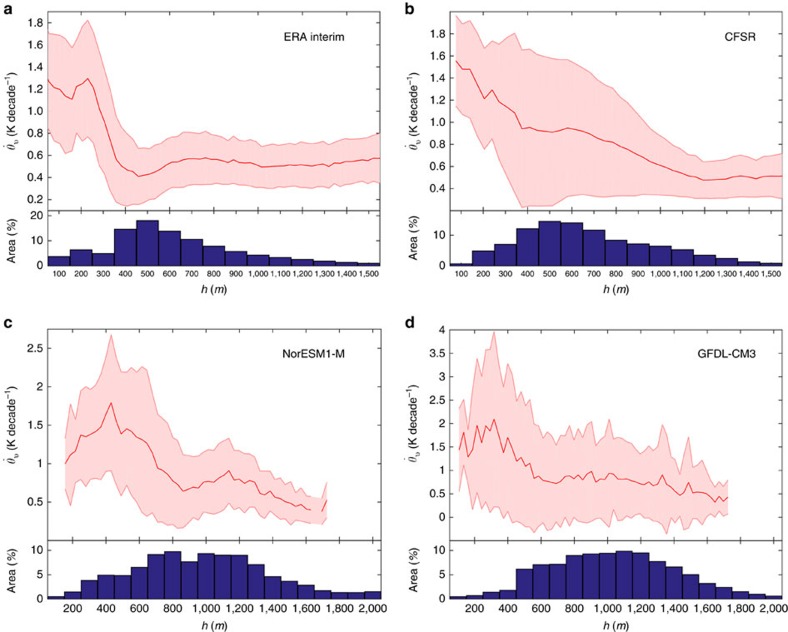
The amplification of temperature trends in shallow boundary layers. The bin mean and s.d. of the inter-annual trend in the virtual potential temperature at a height of 2 m above the ground, as a function of the climatological monthly mean planetary boundary layer depth for (**a**) ERA-Interim reanalysis, (**b**) CFSR reanalysis, (**c**) the Geophysical Fluid Dynamic Laboratories Coupled Model 3 (GFDL-CM3) and (**d**) the Norwegian Earth System Model (NorESM1-M) climate model simulations. The ERA-Interim and CFSR data are taken over the period 1979–2014 and the climate model data from 1979–2005.

Here we have identified the relationship between the boundary layer depth and the trend in *θ*_v_ during the recent warming period, as seen in different re-analyses, and in two state-of-the-art global climate models (Fig. 3). There is a distinctly inverse relationship, where the strongest warming trends are found in shallow boundary layers, and correspondingly low atmospheric heat capacity. The strength of the trends decrease rapidly as we go towards deeper boundary layers and then remain relatively constant across a wide range of boundary layer depths. This amplified temperature trend in the shallowest boundary layers can also be seen in climate models with very different climatologies of the PBL depth. The Norwegian Earth System Model and Geophysical Fluid Dynamic Laboratories Coupled Model 3 have very different climatologies of the PBL depth compared with the re-analyses (Supplementary Fig. 1); both have biases towards deeper PBLs, but they still show a significant correlation between inverse boundary layer depth and the magnitude of trends in *θ*_v_ (*R* = 0.35, *P* < 0.05 and *R* = 0.32, *P* < 0.05, respectively). This process where heat gets trapped in a shallow layer near the surface by stable stratification in the boundary layer has been shown to be one of the dominant causes of Arctic amplification^30^,^31^, but it also has a crucial implication for how we assess the efficacy of different forcings in affecting the SAT. Given the large differences between the climatology of the PBL depth in re-analyses and the global climate models shown here, and the controlling influence the boundary layer depth has on the surface climate^32^, this is clearly an area that requires more attention, especially in future model development.

### Variability in the strength of the boundary layer effect

**Figure 4:**
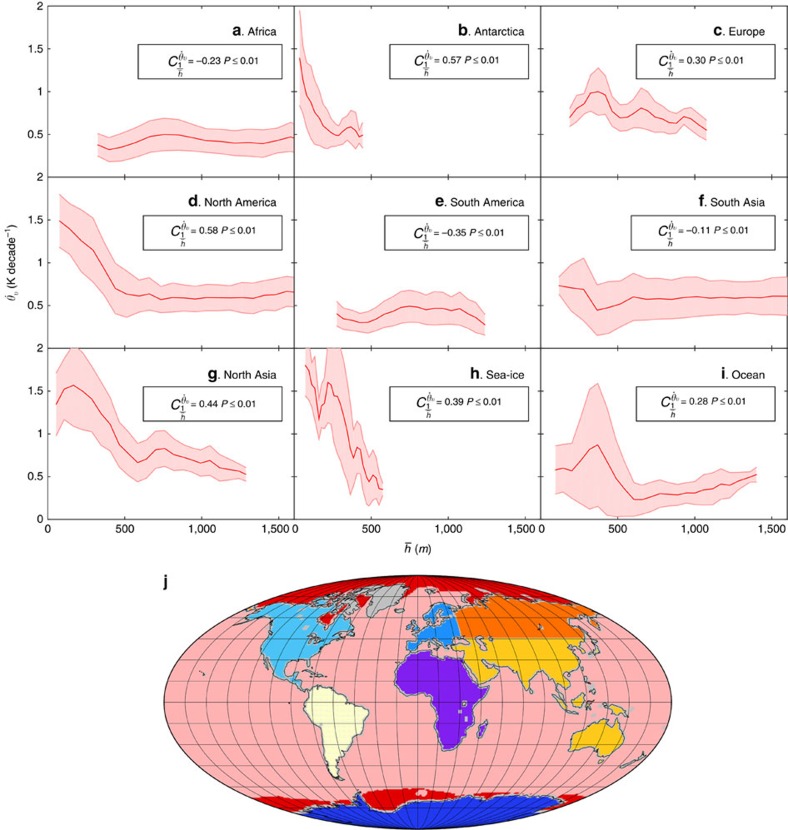
Geographical variations to the planetary boundary layer effect. The inter-annual trend in the virtual potential temperature at a height of 2 m above the ground as a function of the climatological monthly mean planetary boundary layer depth is shown for nine different regions: (**a**) Africa, (**b**) Antarctica, (**c**) Europe, (**d**) North America, (**e**) South America, (**f**) South Asia, (**g**) North Asia, (**h**) Sea-ice and (**i**) Ocean, as illustrated on the (**j**) map of the Earth. The thick red line indicates the bin-mean and the shaded area shows the region of 1 s.d. The correlation between the magnitude of the temperature trends and the inverse boundary layer depth is given for each region.

Owing to its varied climatology (Supplementary Fig. 2), there are some geographical differences as to when the boundary layer depth becomes important in determining the strength of temperature trends. For deep boundary layers the relationship between boundary layer depth and temperature trends is expected to be small, and it is the strength of the local forcing factors themselves, which will principally determine the variations in the rate of warming. However, in shallower boundary layers the strength of temperature trends may be expected to become increasingly dependent on the boundary layer depth. This can be seen in the correlation between the magnitude of temperature trends and the inverse boundary layer depth for different geographical regions (Fig. 4). In high-latitude continental regions such as North America, North Asia and Antarctica, where we frequently get very shallow boundary layers in autumn and winter, there is a strong correlation between inverse boundary layer depth and *θ*_v_ trends. Whereas in more tropical regions such as Africa, South Asia and South America, where cases of shallow boundary layers are less frequent, there is no evidence of this amplification effect.

There is also a strong seasonal variation in the PBL amplification effect: in the boreal spring and summer, the boundary layer is relatively deep and so the amplification effect is relatively weak. However, during the boreal autumn and winter, we can see that the PBL depth is very small over land (Supplementary Fig. 2); thus, during these periods the amplification effect of the PBL depth can become important and should be taken into account. This is why studies that have chosen to focus on the mid-latitudes during the summer seasons have had some success in demonstrating a relationship between a given forcing process and changes to the SAT^9^, whereas more global analysis of the same processes have shown weaker relationships^8^. From the ERA-Interim results, we can see that the amplification effect becomes very apparent for boundary layers less than a few hundred metres (Fig. 3). This is quite common, with PBL depths <400  m occurring >46 % of the time in ERA-Interim. This is a good indication of the fraction of time that the PBL depth becomes important in determining the strength of temperature trends.

### Including the boundary layer effect in signal detection

**Figure 5:**
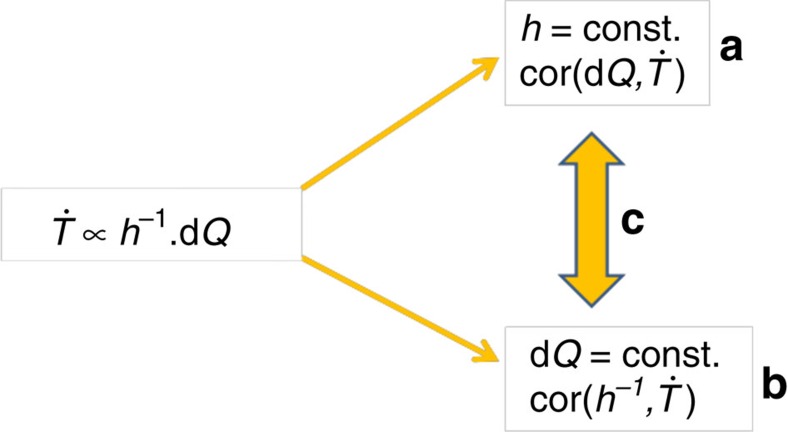
Relationship of proposed co-variability method to existing methodology. Schematic of the proposed co-variability method and the relation to: (**a**) current methodology where variations in the PBL depth, *h*, are neglected and we directly relate surface temperature trends, 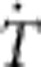, with climate perturbations, *dQ*; (**b**) a uniform climate-forcing perturbation where it is the climatology of the PBL depth, which is the best predictor of temperature trends; and (**c**) intermediary conditions where both the PBL depth and the perturbation in the forcing are significant in determining the temperature response.

One way to account for the PBL depth in the analysis of climate processes is by considering the integrated temperature response within a co-variability framework (Fig. 5). In this framework, the net temperature change is proportional to the time integration of the product of the perturbations in the forcing, *dQ*, with the inverse boundary layer depth, *h*^−1^. In this regard, the conventional methodology is a limit of the co-variability framework, when the variations in the heat capacity can be neglected and we can directly relate perturbations in the forcing to perturbations in the surface temperature (case A, Fig. 5). This is a reasonable approximation only if that forcing is applied solely to deep or weakly varying boundary layers such as the tropical marine boundary layer^17^. The other limit of the co-variability framework occurs when we have a uniformly applied perturbation to the climate forcing (case B, Fig. 5). In this limit, it is the climatology of the boundary layer depth that principally determines the pattern of warming/cooling in response to a perturbation in the surface heating. The enhanced concentration of GHGs is one such example of a near-uniform perturbation in the surface heating and, as such, there is a strong relationship between the inverse boundary layer depth and the strength of temperature trends in re-analysis (Fig. 3), and both within and between global climate models^32^.

**Figure 6:**
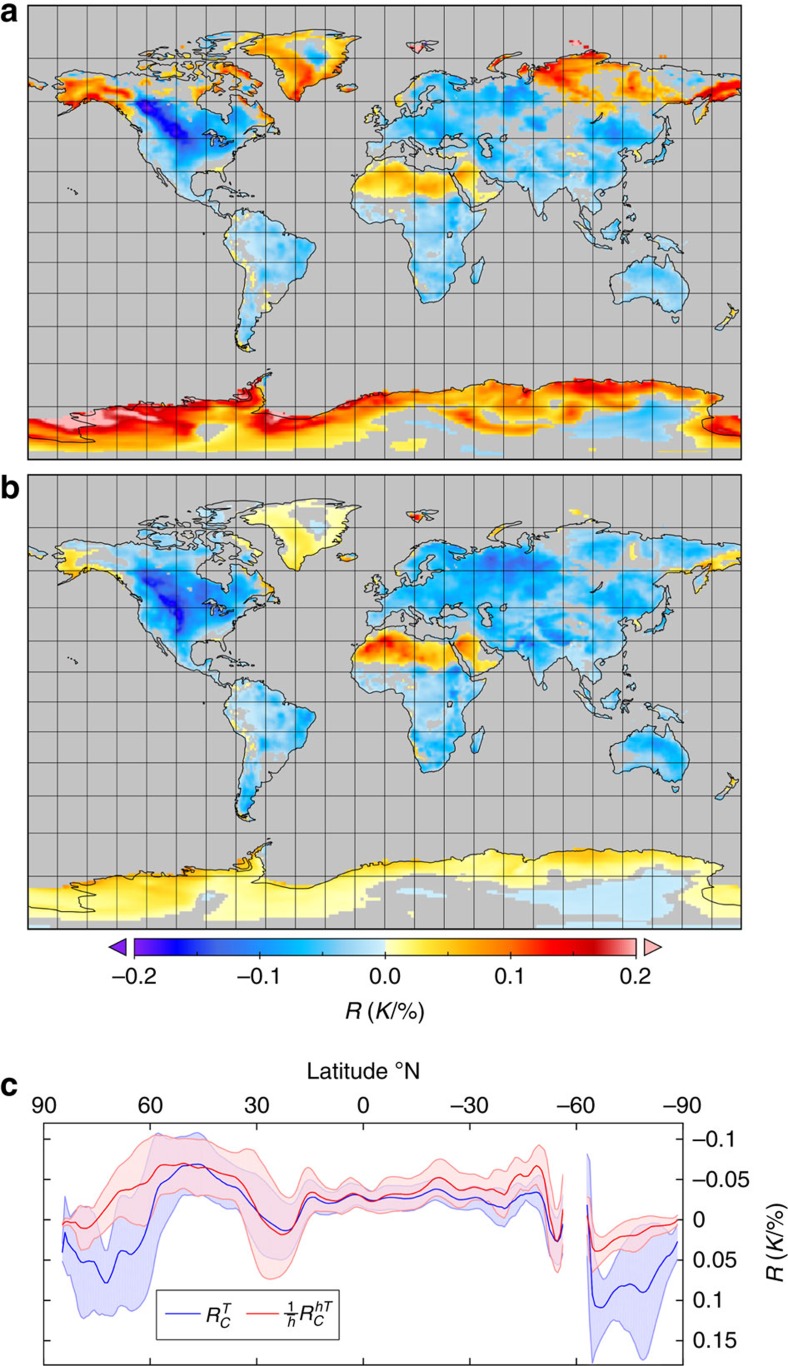
The climate effect of clouds on temperature and heat content. The maps show the slope of the linear regression, *R*, between anomalies in cloud cover and (**a**) anomalies in SAT, and (**b**) anomalies in normalized heat content in K%^−1^ change in cloud cover. We also show (**c**) the bin mean and s.d. of the regression coefficients as a function of latitude.

However, in most cases it is both the PBL depth and the strength of the forcing that will be important in determining the spatial and temporal variations of climate change. In these cases, it is necessary to account for the nonlinear amplification effect of the PBL depth. Let us take the example of the influence of cloud cover on surface temperatures. We expect that an increase in cloud cover during the day will damp incoming solar radiation and thus decrease surface temperatures^9^. However, an increase in cloud cover at night is expected to reduce longwave cooling and hence result in warmer surface temperatures. Thus, the net effect of changes to the cloud cover on the surface climate is determined by the balance between the cooling effect of damped shortwave radiation and the warming effect of reduced longwave cooling. The cooling effect principally applies during conditions with strong surface heating (when the surface energy balance is dominated by shortwave radiation) and applies to deep PBLs, compared with the warming effect that dominates when there is a net longwave cooling and relatively shallow PBLs. Therefore, when we consider the effect of changes in cloud cover on the atmospheric heat content, rather than on the surface temperature, we expect the cooling effect to become more apparent. This can be seen in the regressions of the cloud cover anomalies, 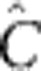, against surface temperature anomalies, 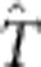, and against normalized atmospheric heat content anomalies, 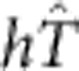 (Fig. 6). If we look at the sensitivity of surface temperature to cloud cover we can see that in the high latitudes the strong winter-time warming effect of increased cloud cover dominates on the inter-annual scale and we get a strong positive relationship, whereas when we consider the effect of cloud cover on heat anomalies we find a more widespread cooling effect of increased cloud cover, even in these high-latitude continental interiors.This may be expected, as the warming effect of positive cloud cover perturbations on the surface temperature that occurs during the winter months only has a small impact on the atmospheric heat content compared with the cooling that occurs in deep PBLs in the summer months. Thus, when we account for variations in the effective heat capacity, we get a significantly stronger damping of atmospheric heat content from increased cloud cover than we found when assessing surface temperatures: the globally averaged overland temperature sensitivity to cloud cover is −12 (±17) × 10^−3^ K %^−1^, compared with a sensitivity of normalized heat content to cloud cover of −32 (± 16) × 10^−3^ K %^−1^. This marks a much clearer signal of an overall cooling effect of increased cloud cover on the surface climate.

## Discussion

What is proposed here is essentially a way of accounting for the fact that processes such as changes to cloud cover, soil moisture and so on introduce perturbations to the heating, but what we measure is the temperature perturbation. It has been shown that we can better understand influences on the climate system by considering the heat content, rather than the temperature, of the components of the climate system^33^; however, the importance of spatial and temporal variations in the heat capacity have largely been neglected. This can be especially important within the diurnal cycle, when the shallow boundary layer depths at night drive strong changes to the nocturnal temperatures^12^, which have a strong impact on vegetation^34^.

Here we have shown that by accounting for the variations in the effective heat capacity of the atmosphere we can more accurately assess how a given process influences the surface climate. This allows us to directly compare, in an apples-to-apples manner, the relative importance of different processes in determining the overall response of the climate system to perturbations in the climate forcing. This is also critical for model-model and model-observation comparisons of climate forcing processes. There is a very varied climatology of the PBL in different climate models and re-analysis (Fig. 3) (refs 32, 35, 36) and so these models can exhibit very different temperature responses, given the same change in the climate forcing.

## Methods

### Processing of re-analysis data

We obtained the monthly mean time series of air and dew-point temperature at a height of 2  m above the ground, boundary layer depth and sea-ice concentration from the European Centre for Medium-range Weather Forecasts website for the full period of available data, 1979–2014. The air and dew-point temperature were then used to calculate the virtual potential temperature at a height of 2  m above the ground, *θ*_v_. The boundary layer depth in the ERA-Interim model is calculated using an iterative bulk-Richardson method, which scans upwards from the lowest model level and interpolates between model levels to find the height at which the bulk Richardson number first exceeds the critical Richardson number, *Ri*_cr_, taken to be 0.25. Although the bulk-Richardson method has been shown to work well in both stable and unstable stratification^35^, this definition does create some problems in tropical regions, including parts of South America, Africa and South Asia. The estimation of the boundary layer depth from different methods shows great variation in these regions of high surface humidity and strong convection, and so the ERA-Interim PBL depth is not necessarily reflective of the vertical extent of turbulent mixing^36^. This is likely to be the reason for the lack of a clear signal of shallow boundary layer amplification of temperature trends in these regions (Fig. 4).

The models used in both re-analysis and GCMs also tend to be biased towards producing deeper-than-observed boundary layers, especially under stably stratified conditions^35^,^37^. This is probably due to our limited understanding of geophysical turbulence under stable stratification and the limited applicability of current turbulence parameterization schemes to strongly, stably stratified conditions^37^. This bias has been shown to lead to an underestimation of the SAT response to climate forcing^32^.

The sea-ice concentration was used to define the region ‘sea ice' in Fig. 4. The requirement for being considered over sea ice was that the minimum concentration for a location in a given month be >80% in all years.

The monthly mean time series of air temperature and specific humidity at a height of 2  m above the ground, the boundary layer depth and sea-ice concentration were obtained from the National Center for Atmospheric Research website. The boundary layer depth in this model is calculated in a similar way as to that in ERA-Interim, using the bulk-Richardson formulation and the same critical Richardson number.

### Analysis of climate model data

To calculate the boundary layer depth in these models, we used a bulk Richardson method. This was chosen due to the limits of data availability for other methods, for example, no information on local gradients was available, which would be required for a flux-Richardson method, and the vertical resolution in these models is too coarse for any of the profile-based methods. The bulk-Richardson method has also been shown to be the most robust across a wide range of thermal stability and is consistent with the method used in the re-analyses.

First, we obtained the 6-hourly resolution three-dimensional fields of the wind components, humidity and temperature, and the 3-hourly resolution surface temperature, pressure and humidity for the period 1979–2005 for Norwegian Earth System Model and Geophysical Fluid Dynamic Laboratories Coupled Model 3 from the British Atmospheric Data Centre archive. The temperature and humidity fields were used to calculate the virtual potential temperature at each model level and at the surface using the definition 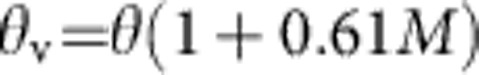, where *θ* is the potential temperature and *M* is the water–vapour mixing ratio. The thickness of each model level, *Z*, was calculated using a hydrostatic assumption, such that 
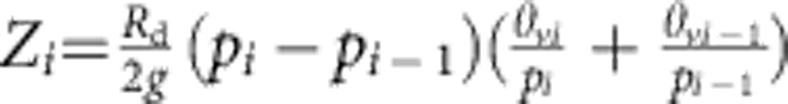
, where *i* is the index of the model level with the surface being *i* = 0, *θ*_v_ is the virtual potential temperature, *p* the pressure, *R*_d_ the dry gas constant ( 287.06 J   kg^−1^  K^−1^) and *g* the surface gravity ( 9.807 m  s^−2^). The geometric height of each level was then calculated by the summation of the thickness of the lower levels. The bulk Richardson number at each pressure level was calculated using the difference between that level and the surface: 
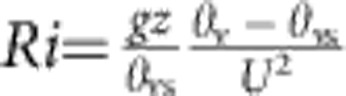
, where *g* is the surface gravity, *z* is the height above the surface, *θ*_v_ is the virtual potential temperature at height *z*, *θ*_vs_ is the virtual potential temperature at a height of 2  m above the surface and *U* is the wind speed at height *z*. We then scanned upwards from the lowest level above the surface and linearly interpolated between levels, to find the first height at which the bulk Richardson number exceeded the critical value, *Ri*_cr_. We also applied a requirement that the PBL depth should be >10  m and <4  km: as this was an automated method, these constraints were necessary to avoid cases where the routine returned unphysical PBL depths.

### Statistical methodology

The correlations given in Fig. 4 are the area-weighted spatial correlations between the monthly mean inverse boundary layer depth and the inter-annual trend in the monthly mean virtual potential temperature at a height of 2 m above the ground. The *P*-values were computed to test against the null hypothesis of zero correlation using a Student's *t* distribution for a transformation of the correlation.

The regression coefficients in Fig. 6 were determined from a least-square, best-fit linear regression between the two variables under consideration. These were the cloud-cover anomalies against the anomalies in *θ*_v_ and the cloud cover anomalies against the normalized heat anomalies, 
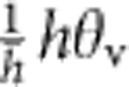
, where 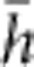 is the area-weighted, climatological mean boundary layer depth. Anomalies were calculated by removing the climatological mean of each month from the time series using the full period of the data, 1979–2014.

### Code availability

The Matlab code used to generate the PBL depth and virtual potential temperature data sets discussed in this work have been archived by the authors and are available on request from the corresponding author, R.D. Richard.davy@nersc.no).

### Data availability

All the model data used in this study is publicly available from the respective host institutions or archives. The ERA-Interim reanalysis data may be obtained from http://dx.doi.org/10.5065/D6CR5RD9. The CFSR reanalysis data are available from http://dx.doi.org/10.5065/D69K487J and the climate model data are available from https://esgf-data.dkrz.de/search/cmip5-dkrz/.

## Additional information

**How to cite this article:** Davy, R. & Esau, I. Differences in the efficacy of climate forcings explained by variations in atmospheric boundary layer depth. *Nat. Commun.* 7:11690 doi: 10.1038/ncomms11690 (2016).

## Supplementary Material

Supplementary Information Supplementary Figures 1-2

## Figures and Tables

**Figure 1:**
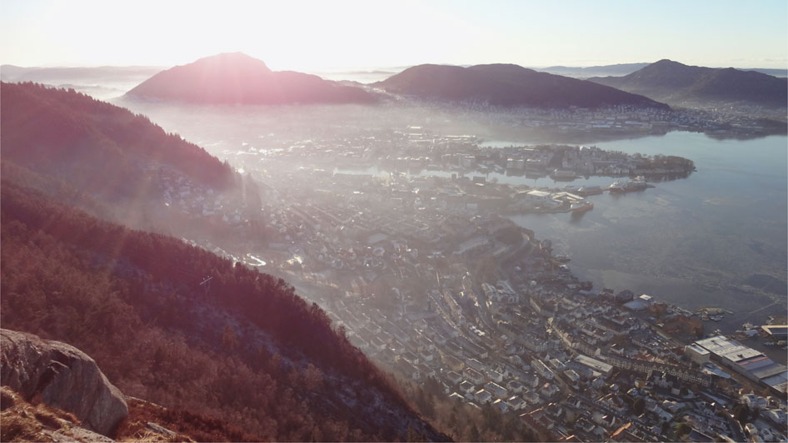
Visualization of the separation of the stable boundary layer. Water vapour in the planetary boundary layer over Bergen, Norway, highlights the trapping of air in the urban boundary layer. Courtesy, T. Wolf, NERSC.

**Figure 2:**
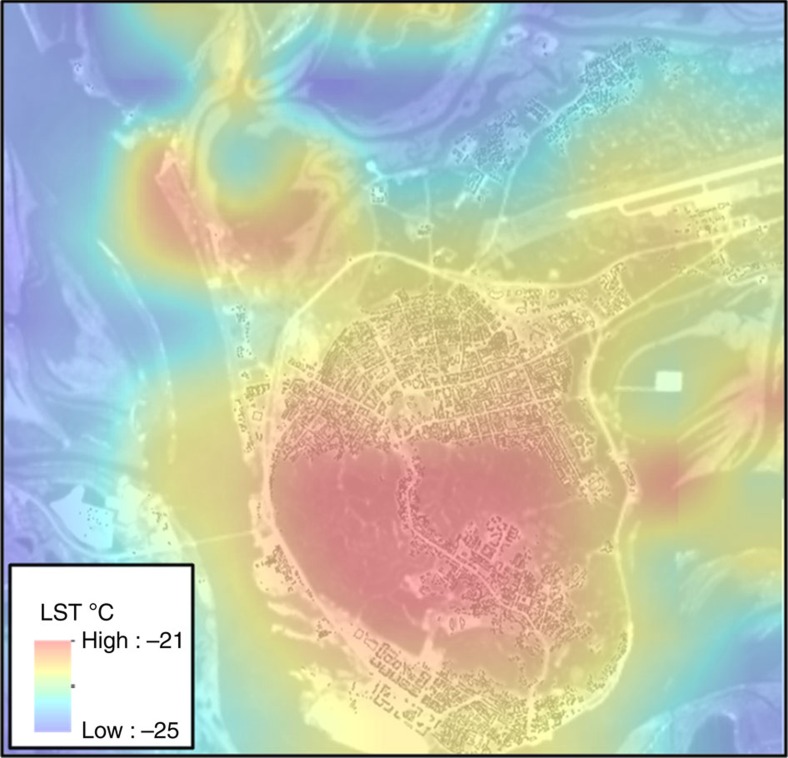
Satellite observation of the urban heat island effect. The urban heat island effect, as seen in 15-year climatological mean winter-time land surface temperatures (LST) over Khanty-Mansiysk, Russia, from MODIS observations. Courtesy V. Miles, NERSC.
