# Supplementary material for: Differences in the efficacy of climate forcings explained by variations in atmospheric boundary layer depth
Source: Nat Commun. 2016 May 25;7:11690. doi: 10.1038/ncomms11690 (PMC4894963; doi:10.1038/ncomms11690)
Supplement: Supplementary Information — Supplementary Figures 1-2 [file ncomms11690-s1.pdf]

Supplementary Figures.

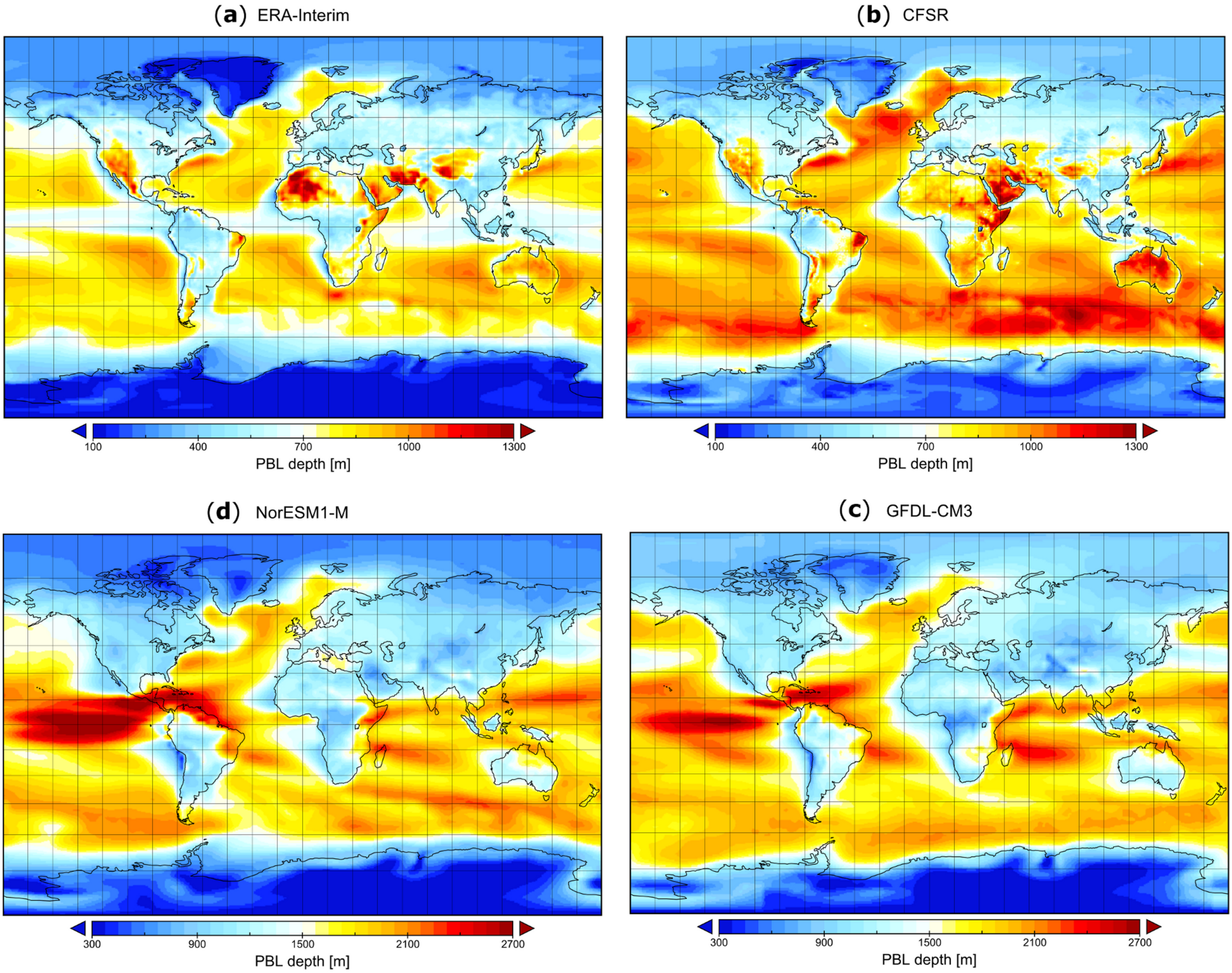

Supplementary Figure 1 | The climatology of the boundary layer depth in climate models and reanalyses. The climatological, annual-mean boundary layer depth from (**a**) ERA-Interim reanalysis, (**b**) CFSR reanalysis, (**c**) the GFDL-CM3, and (**d**) the NorESM1-M climate model historical simulations.

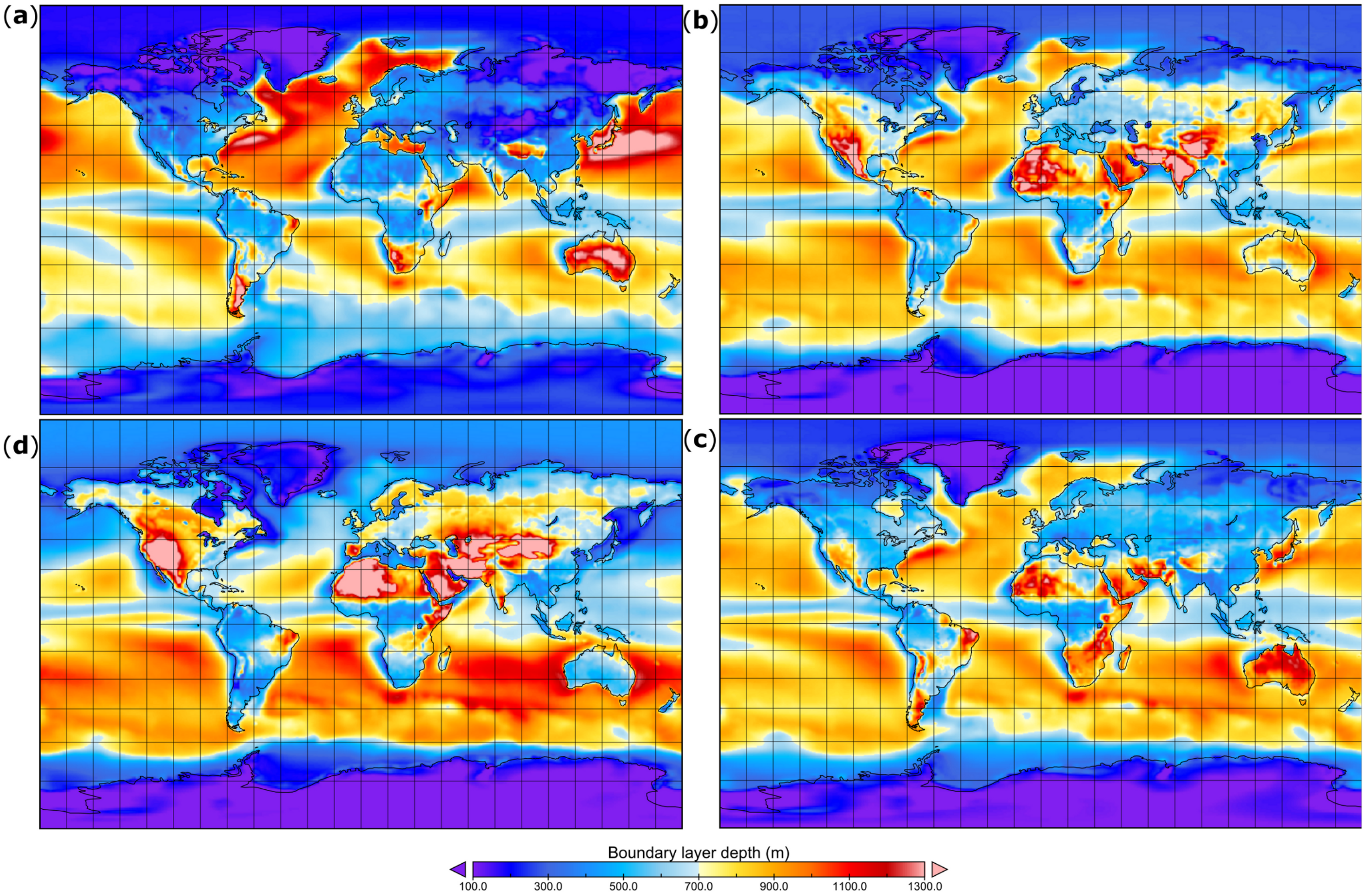

Supplementary Figure 2 | The seasonal cycle in boundary layer depth. The climatological-mean boundary layer depth for the months: (**a**) Dec-Jan-Feb, (**b**) Mar-Apr-May, (**c**) Sep-Oct-Nov, and (**d**) Jun-Jul-Aug. The data are taken from ERA-Interim over the period 1979-2014.
